# Transcutaneous auricular vagus nerve stimulation for pediatric epilepsy: study protocol for a randomized controlled trial

**DOI:** 10.1186/s13063-015-0906-8

**Published:** 2015-08-21

**Authors:** Wei He, Xiao-Yu Wang, Li Zhou, Zhi-Mei Li, Xiang-Hong Jing, Zhong-Li Lv, Yu-Feng Zhao, Hong Shi, Ling Hu, Yang-Shuai Su, Bing Zhu

**Affiliations:** Institute of Acupuncture and Moxibustion, China Academy of Chinese Medical Sciences, Beijing, 100700 China; Hubei University of Chinese Medicine/Innovation Center of Preventive Treatment by Acupuncture and Moxibustion, Wuhan, 430061 China; Beijing Tian Tan Hospital, Capital Medical University, Beijing, 100050 China; Beijing Children Hospital, Capital Medical University, Beijing, 100045 China; Clinical Evaluation Center, Institute of Basic Research in Clinical Medicine, China Academy of Chinese Medical Sciences, Beijing, 100700 China

**Keywords:** Transcutaneous auricular vagus nerve stimulation, Pediatric epilepsy, Randomized controlled trial, HRV

## Abstract

**Background:**

Recently, clinical observations reported the potential benefit of vagus nerve stimulation (VNS) for pediatric epilepsy. Transcutaneous auricular vagus nerve stimulation (ta-VNS) is a newer non-invasive VNS, making it more accessible for treating pediatric epilepsy, yet there is limited clinical evidence for its effectiveness.

**Methods/Design:**

A three-center, randomized, parallel, controlled trial will be carried out to evaluate whether ta-VNS improves pediatric epilepsy. Pediatric patients aged 2 to 14 years with epilepsy will be recruited and randomly assigned to transcutaneous auricular vagus nerve stimulation (ta-VNS) group, transcutaneous auricular non-vagus nerve stimulation (tan-VNS) group, and control group with a 1:1: sqrt(2) allocation, as per a computer generated randomization schedule stratified by study center using permuted blocks of random sizes. We will use Zelen’s design, in which randomization occurs before informed consent. Patients in the stimulation groups will receive tan-VNS or ta-VNS three times a day for 6 months. Patients in the control group will not be provided with any stimulation during the 6 months. The guardians of the patients are required to keep a detailed diary to record the data. Outcome assessment including seizure frequency, electroencephalogram (EEG), heart rate variability (HRV) analysis, quality of life (QOL) and adverse events will be made at baseline and 2, 4 and 6 months after ta-VNS initiation. The seizure frequency and adverse events will be followed up at 1 year and 1.5 years after ta-VNS initiation.

**Discussion:**

Results of this trial will help clarify whether ta-VNS treatment is beneficial for pediatric patients, and will make clear whether the anticonvulsive effect of ta-VNS is correlated with the improvement of sympathovagal imbalance.

**Trial registration:**

Clinical Trials Identifier: NCT02004340. Registration date: 13 November 2013.

**Electronic supplementary material:**

The online version of this article (doi:10.1186/s13063-015-0906-8) contains supplementary material, which is available to authorized users.

## Background

Vagus nerve stimulation (VNS) has been approved by US Food and Drug Administration (FDA) as a complementary treatment for partial refractory epilepsy in patients aged no less than 12 years, since 1997 [1]. Recently, clinical observations indicated the potential benefit of VNS for pediatric epilepsy. In a retrospective review of patients undergoing VNS insertion, it was suggested that VNS is a safe and effective adjuvant therapy for children under 12 years old, with over half reporting significant benefit [2]. Clinical results reported by the Pediatric VNS Study Group showed a median reduction in seizure frequency of 42 % after 18 months of intermittent stimulation of the left vagal nerve [3].

Recently, newer non-invasive VNS has improved the safety and tolerability of VNS, making it more accessible and facilitating further investigations across a wider range of uses [4]. Transcutaneous auricular vagus nerve stimulation (ta-VNS) at the innervation area of the auricular branch of the vagus nerve (ABVN) has been utilized in the treatment of epilepsy. In a pilot study of adult patients, after 24 weeks’ treatment of ta-VNS, 8/47 patients were seizure-free; 19/47 patients had reduced seizure frequency [5]. It has also been reported that ta-VNS can effectively reduce the frequency of seizures and improve the patient’s quality of life in adult epilepsy patients [6]. In our pilot trial of ta-VNS for pediatric epilepsy, mean reduction in seizure frequency relative to baseline was 54.13 % at the end of 24 weeks’ treatment [7]. Yet no randomized controlled trial (RCT) has been performed on the safety and efficacy of ta-VNS for patients with pediatric epilepsy.

Autonomic imbalance has been found to be accompanied with epilepsy. An increased sympathetic activity and reduced parasympathetic activity were observed in patients with epilepsy [8, 9]. In children with refractory epilepsy, parasympathetic activity is lower in comparison with healthy controls [10]. A systematic review confirmed a sympathovagal imbalance in epilepsy, as shown by lower high-frequency (HF) power spectrum, standard deviation of normal-to-normal interval (SDNN) and root mean square of successive differences (RMSSD) values when compared to controls [11].

In the present study, we use transcutaneous auricular non-vagus nerve stimulation (tan-VNS) as a sham comparator, waiting list group as a control. We aim to investigate the safety and efficacy of ta-VNS in treating pediatric patients with epilepsy in a multicenter RCT, and determine whether the anticonvulsive effect of ta-VNS is correlated with the improvement of sympathovagal imbalance.

## Methods

### Study design

A multicenter randomized, parallel, controlled trial is currently being performed. Pediatric patients will be included from three centers, including Beijing Tiantan Hospital and Beijing Children Hospital affiliated to Capital Medical University, and Acupuncture Hospital affiliated to the Institute of Acupuncture and Moxibustion, China Academy of Chinese Medical Sciences. The protocol has been approved by the Clinical Trial Ethics Committee of the Institute of Acupuncture and Moxibustion, China Academy of Chinese Medical Sciences (number: 2012030101) and registered on ClinicalTrials.gov protocol registration system (Clinical Trials Identifier: NCT02004340). Informed consent will be sought from all the guardians of the participants.

**Fig. 1:**
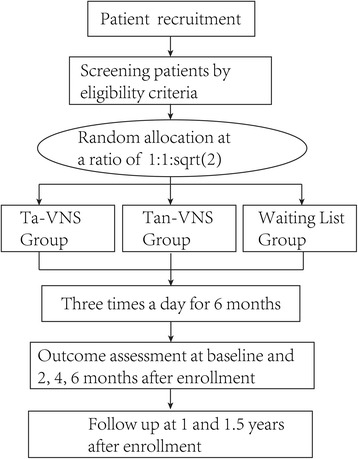
Trial flow chart

At the beginning of the enrollment, participants in the control group will be given a diary book to record the data, including the frequency of seizures and adverse events. Participants in the stimulation groups will be given a ta-VNS instrument and a diary book to record the frequency of seizures, usage of instruments and side-effects or adverse events. Patients or their guardians will be required to keep a detailed diary. This trial will include a 6-month treatment period and a 1.5-year follow-up period. Outcome assessment of ta-VNS will be made at baseline and 2, 4 and 6 months, 1 year and 1.5 years after ta-VNS initiation (Fig. 1).

### Inclusion criteria

Participants will be included if they meet all of the following conditions: 1) diagnosis of epilepsy, and aged between 2 and 14 years old; 2) the number and dose of the medication has been kept constant no less than 2 months before intervention; 3) the number of epileptic seizures is no less than eight in 2 months; 4) the patients or their guardians can count the frequency of the seizures and finish the study; and 5) the guardians can understand the mechanism of the ta-VNS treatment, voluntarily agree with the study protocol and sign written informed consents.

### Exclusion criteria

Participants will be excluded if they meet any of the following conditions: 1) the patient is receiving VNS therapy; 2) the patient is accompanied with progressive central nervous disease; 3) the patient has severe heart, liver or blood disease; or 4) previous participation in this study.

### Randomization and blinding

Participants will be randomly assigned to either one of the two stimulations or the control group with a 1:1: sqrt(2) allocation, as per a computer generated randomization schedule stratified by the study center using permuted blocks of random sizes. The block sizes will not be disclosed, to ensure concealment. We will use Zelen’s design, in which randomization occurs before informed consent. Those patients in the control group (n = 42) receiving standard care will not be consented for participation in this study. They will be informed in an observational study with questionnaires to provide the same measurements, except for treatment, as that in the treatment groups for free, and thus blinded. Other participants who accept the intervention will provide further informed consents, in which they will be informed to have the possibility of receiving either one of the two stimulations according to computerized randomization schedule. The two stimulation groups will include the tan-VNS group (n = 30) and the ta-VNS group (n = 30). Participants in the control group and stimulation groups are thus blinded.

The randomization sequence will be generated using SPSS v.17.0 software (IBM, Armonk, NY, USA) and distributed in a sealed envelope to the doctor. The researchers will not be permitted to change it after the patients are randomized. Employees outside the research team will feed original data into the computer in separate datasheets so that the researchers can analyze data without having access to information about the allocation. The evaluation of the curative effect and the statistical analysis will be evaluated by a third party in the clinical evaluation center of the China Academy of Chinese Medical Sciences who will not know the grouping and patients. In this clinical trial process, the patients, statisticians and the assessor for statistical data and outcomes will be blind to treatment allocation. Allocation concealment will not be exposed until the final data analysis report is completed.

### Interventions

For patients in the ta-VNS group, transcutaneous stimulation will be performed at the bilateral auricular concha with two pairs of stimulation electrode clips via the ear vagus nerve stimulator (TENS-200, Suzhou, Jiangsu, China) [7]. The stimulation electrodes are made of conductive rubber, with a diameter of 5 mm. The anode electrode is placed on the concha cavity and the cathode electrode is placed on the cymba concha. For patients in the tan-VNS group, transcutaneous stimulation will be performed at the marginal area of the auricle, which is not innervated by the ABVN. The parameters of the stimulation are as follows: frequency of 20 Hz; intensity of 1.0 mA; duration of 30 min; three times a day; and for a total of 6 months. Professional technicians will train the patients or their guardians about the use of the stimulators; patients then take the stimulators home and initiate the stimulators as needed.

For the control group, no transcutaneous stimulation is provided during the 6 months.

### Outcome measures

#### Primary outcomes

Seizure frequency: the baseline seizure frequency and the seizure frequency after 2 months, 4 months and 6 months of enrollment will be counted according to the patient’s seizure diary. Seizure outcomes will be expressed with a modified Engel scale [12].

#### Secondary outcomes

Video electroencephalogram (EEG): video EEG of each patient will be recorded for 2 hours before ta-VNS initiation and at the end of ta-VNS, respectively.

Heart rate variability (HRV): HRV of each patient will also be recorded before ta-VNS initiation and at the end of ta-VNS, respectively.

Health-related quality of life (QOL): parent’s perceptions of the child’s general behavioral problems will be quantified by using the total score of Achenbach’s Child Behavior Checklist (CBCL) [13]. The CBCL for ages 1.5–5 years and for ages 6–18 years are 99-item and 118-item questionnaires, respectively, and assesses behavioral problems in children. The guardians of the patients will be asked to rate whether a behavioral problem is present or not in the child and to what degree.

Side-effects or adverse outcomes: information about adverse outcomes will be recorded in the diary by the patient or their guardian. If a possible side-effect or adverse reaction occurs, the patient or their guardian should discontinue the stimulation and contact the doctor who will make a judgment. If serious side-effects occur, the doctor can then unblind the participant and give the patient post-trial care. All side-effects will be recorded and reported.

Detailed information about the whole schedule is summarized in Table 1.

**Table 1:** Detailed schedule of the protocol

Schedule	Baseline	Treatment phase			Follow-up phase	
	0 months	2 months	4 months	6 months	1 year	1.5 years
Patients						
Informed consent	×					
Medical history	×					
Physical examination	×					
Randomization	×					
Intervention						
ta-VNS group	Transcutaneous stimulation at the auricular concha					
tan-VNS group	Transcutaneous stimulation at marginal area of the auricle					
Comparison						
Control group	No transcutaneous stimulation is provided					
Outcomes						
Seizure frequency	×	×	×	×	×	×
EEG	×			×		
HRV	×			×		
QOL	×			×		
Safety						
Adverse events	×	×	×	×	×	×

The symbol ‘x’ is used as a check mark

*EEG* electroencephalogram; *HRV* heart rate variability; *QOL* quality of life; *ta-VNS* transcutaneous auricular vagus nerve stimulation; *tan-VNS* transcutaneous auricular non-vagus nerve stimulation

### Quality control and trial monitoring

Before the initiation of the trial, the researcher will formulate an investigator’s brochure, standard operating procedures and detailed research plan. All the staff should participate in special training, including enrolling patients, completing the case report form and usage of the stimulator. The data monitoring committee has been established, which is independent from the sponsor and competing interests. Monitors will check case report forms at the participating hospitals once a month. Drop-outs and withdrawals (and the reasons) from the study will be fully documented throughout. All the data, including the case report forms and electronic documents, will be kept by special staff. The trial sponsor (BZ) has access to the final trial dataset.

The final report will follow the Consolidated Standards of Reporting Trials (CONSORT) extension guideline for non-pharmaceutical interventions. In order to minimize 6 months’ attrition, first we will enrol patients whose guardians can understand the mechanism of the ta-VNS treatment and our previous results. Second, in the consent inform, the participant and their guardian will be informed that the patient can get doctor consultation during the whole study period. They can also get a physical examination (EEG and HRV) and an auricular vagus nerve stimulator for free when they complete the study. The doctor will keep in touch with the patients once a month by phone or email. Additional file 1 describes the protocol in more detail.

### Statistical analysis

The intention-to-treat population will be defined as the participants who are randomized and received at least one treatment session. An interim analysis will be performed on the primary endpoint when 50 % of patients have been randomized and have completed the 1.5 years’ follow-up. The interim analysis will be performed by an independent statistician, blinded for the treatment allocation. All statistical calculations will be performed using SPSS v.17.0. Comparison of seizure frequency, HRV and QOL between baseline and after treatment will be analyzed by paired-samples t-test. The Kolmogorov-Smirnov test will be used to evaluate if groups fit normal distributions. Normally distributed groups will be analyzed by parametric tests. Comparisons between the three groups will be analyzed by one-way ANOVA followed by Student–Newman–Keuls or Dunnett’s T3 post-hoc tests. Non-normally distributed groups will be analyzed by Mann–Whitney’s test. The factors that affect curative effect will be analyzed with the multinomial logistic regression method. A *P* value <0.05 will be considered of statistical significance.

## Discussion

Vagus nerve stimulation (VNS) is effective in refractory epilepsy and depression, and is being investigated in heart failure, headache, gastric motility disorders and asthma [4]. In addition to the suppression of seizure frequency, QOL of the pediatric patients with epilepsy also improved by VNS [14]. In patients with drug-resistant focal epilepsy, VNS reduces cardiac electrical instability, potentially by improving autonomic imbalance in favor of parasympathetic dominance [15]. Nonetheless, adverse events are generally associated with implantation or continuous on-off stimulation, including infection, bradycardia, vocal cord paresis, voice alteration, cough, headache, dyspnea, pharyngitis and pain [4].

As the only peripheral branch of the vagus nerve, the ABVN has been suggested as a target of transcutaneous vagus nerve stimulation (t-VNS). In comparison with VNS, t-VNS is more economic with less side-effects. Particularly for pediatric patients, transcutaneous stimulation is preferred. In addition to epilepsy, ta-VNS is now being investigated in anti-atrial fibrillation [16], depression [17] and tinnitus [18]. It has been reported that in healthy participants, active t-VNS significantly increased HRV and reduced sympathetic nerve outflow [19]. Yet the relationship between the anticonvulsive effect of ta-VNS and the improvement of autonomic imbalance needs to be further investigated.

Although the mechanism of the anticonvulsive effect of VNS is unclear, it has been suggested to be mediated via vagal afferent projections to the nucleus tractus solitarius (NTS), then via pathways from the NTS to other brain structures which correlate with the pathogenesis of epilepsy [20]. Animal studies have shown an anatomical relationship between the ABVN and the NTS, and ta-VNS significantly suppressed epileptiform activity in EEG traces via increasing the firing rates of the neurons of the NTS [21]. Studies in humans have shown that auricular conchae stimulation produced significant activation of the central vagal projections including widespread activity in the ipsilateral NTS [22]. The NTS receives and integrates visceral autonomic afferent information, which indicates the possibility of the NTS participating in the anticonvulsive effect of ta-VNS.

Currently, there are no RCTs investigating ta-VNS for pediatric patients with epilepsy. Here, we designed this study to conduct a RCT to examine the potential effect of ta-VNS and its possible mechanism.

## Trial status

This trial is currently recruiting participants. To date (15 February 2015), 35 participants have been recruited.

## Additional file

Additional file 1:
**Standard Protocol Items: Recommendations for Interventional Trials (SPIRIT) 2013 checklist.** The protocol adheres to the SPIRIT guidelines. (DOCX 23 kb)

## Abbreviations

ABVN: Auricular branch of the vagus nerve
ANOVA: Analysis of variance
CBCL: Child Behavior Checklist
CONSORT: Consolidated Standards of Reporting Trials
EEG: Electroencephalogram
FDA: US Food and Drug Administration
HRV: Heart rate variability
NTS: Nucleus tractus solitarius
QOL: Quality of life
RCT: Randomized controlled trial
RMSSD: Root mean square of successive differences
SDNN: Standard deviation of normal-to-normal interval
tan-VNS: Transcutaneous auricular non-vagus nerve stimulation
ta-VNS: Transcutaneous auricular vagus nerve stimulation
t-VNS: Transcutaneous vagus nerve stimulation
VNS: Vagus nerve stimulation
